# Influence of the requirement for abdominal pain in the diagnosis of irritable bowel syndrome with constipation (IBS-C) under the Rome IV criteria using data from a large Japanese population-based internet survey

**DOI:** 10.1186/s13030-018-0137-9

**Published:** 2018-12-05

**Authors:** Masanori Kosako, Hiraku Akiho, Hiroto Miwa, Motoyori Kanazawa, Shin Fukudo

**Affiliations:** 1 Japan-Asia Clinical Development 1, Development, Astellas Pharma Inc., 2-5-1, Nihonbashi-Honcho, Chuo-ku, Tokyo, 103-8411 Japan; 2 Former employee of Astellas Pharma Inc., Tokyo, Japan; 3 Division of Gastroenterology, Department of Internal Medicine, Hyogo College of Medicine, Nishinomiya, Japan; 4 Department of Behavioral Medicine, Tohoku University Graduate School of Medicine, Sendai, Japan

**Keywords:** Irritable bowel syndrome (IBS), Constipation, Rome III, Rome IV, Abdominal bloating, Stress, Food, Epidemiology

## Abstract

**Background:**

Rome III was revised to Rome IV in May 2016. One important change in the Rome IV criteria is that abdominal pain must be present for a diagnosis of irritable bowel syndrome (IBS). Under Rome III, in contrast, patients with abdominal discomfort only could be diagnosed with IBS, but these cases under Rome IV are now classified as unspecified functional bowel disorder (FBD). In a simple comparison of Rome III and Rome IV, it is unclear whether this difference reflects the influence of symptomatic frequency or the presence of abdominal pain. In particular, the influence of abdominal pain restriction on the diagnosis of IBS with predominant constipation (IBS-C) in the Rome IV criteria is largely unknown.

**Methods:**

We reclassified subjects from a Japanese internet survey experiencing abdominal pain or discomfort at least one day each week as surrogate Rome III IBS-C subjects. Among them, we then reclassified subjects experiencing abdominal pain as surrogate Rome IV IBS-C subjects and subjects not experiencing abdominal pain as surrogate Rome IV FBD subjects. Symptoms were quantified and compared between the two groups.

**Results:**

The surrogate Rome IV IBS-C subjects felt a significantly higher degree of anxiety in their daily lives (*p* < 0.001) compared with the surrogate Rome IV FBD subjects. The combined female and 20–49 years surrogate Rome IV IBS-C subjects felt a higher degree of anxiety in their daily lives (*p* < 0.05) than the respective Rome IV FBD subjects.

**Conclusions:**

These results suggest that female IBS-C patients aged 20–49 years with abdominal pain in Rome IV have more anxiety than those without abdominal pain in Rome III. Changes in the diagnostic criteria from Rome III to Rome IV will better identify candidates for the biopsychosocial approach.

**Trial registration:**

Although this survey was an anonymous internet survey, we obtained informed consent for the study as an online response. The disclosure of this study was approved by the Ethics Committee of Tohoku University Graduate School of Medicine (approval number: 2015–1-405).

## Background

Irritable bowel syndrome (IBS) has multiple pathophysiological factors, including abnormalities of gastrointestinal motility, viscerosensory hypersensitivity, brain-gut interaction, infection of the gastrointestinal tract, and psychosocial factors [1, 2]. The brain-gut relation can be aggravated by psychosocial stress, and this is also an important factor in functional gastrointestinal disorders (FGIDs), including IBS [3]. The excitation of autonomic efferent neurons, neuroendocrine secretion, and sensitization of afferent neurons under stress causes abnormalities in gastrointestinal motility and visceral hypersensitivity, and may further aggravate perceived stress [4]. Because this vicious cycle is repeated, the symptoms of IBS can persist for a long period of time. It is important to understand the disease from a biopsychosocial perspective.

Many diagnostic criteria for IBS have been proposed since the publication of the Manning criteria [5]. The momentum to create guidelines for diagnosis and treatment of IBS increased after the symposium on IBS during the World Congress of Gastroenterology held in 1984, and the Rome (I) criteria for IBS were proposed by a global working group in 1989 [6]. As the understanding of functional gastrointestinal disease (FGID) progressed, more exacting criteria were developed, and the Rome II criteria [7] was released in 1999. Rome II was revised to Rome III [2] in 2006, which was used worldwide as the international diagnostic criteria for FGID. In Japan also, the Rome III diagnostic criteria became the mainstream criteria for the diagnosis of IBS. In April 2014, guidelines for the clinical management of IBS were issued by the Japanese Society of Gastroenterology [8]. Rome III was revised to Rome IV in May 2016 [9], which includes new chapters on multicultural differences, age-gender-women’s health, the intestinal microenvironment, biopsychosocial issues, and centrally mediated disorders [10]. One important change in the Rome IV criteria for IBS is that abdominal pain must be present for a diagnosis of IBS [9]. Abdominal discomfort, included in the diagnostic criteria for IBS in Rome III [2], is no longer considered diagnostic for IBS; specifically, while patients with abdominal discomfort only could be diagnosed with IBS based on Rome III, they are classified as having unspecified functional bowel disorder (FBD) based on Rome IV criteria [9]. In a simple comparison of Rome III and Rome IV, it is unclear whether this difference reflects the influence of symptomatic frequency (Rome IV: ≥ 1 day per week, Rome III: ≥ a few days per month) or the presence of abdominal pain (Rome IV: abdominal pain, Rome III: abdominal pain or discomfort). In particular, the influence of the abdominal pain restriction on the diagnosis of IBS-C under the Rome IV criteria is largely unknown.

Our previous internet survey of IBS subjects showed that the prevalence of IBS-C according to Rome III was 2.8% and that abdominal bloating was the most bothersome symptom. IBS-C subjects felt a higher degree of anxiety in their daily lives than control subjects [11]. Using the database developed in that survey, we further reclassified subjects with abdominal pain as surrogate Rome IV IBS-C subjects, and hypothesized that the surrogate Rome IV IBS-C subjects felt a higher degree of anxiety in their daily lives than the surrogate Rome IV FBD subjects, who did not have abdominal pain. This is because the change in abdominal pain is considered to be attributable to affective disturbances and negative emotions [12]. In the present study, we tested this hypothesis by analyzing the data and investigating the influence of abdominal pain restriction on the diagnosis of IBS-C in the Rome IV criteria.

## Methods

This study used the same database as that published in our previous study [11] but used a different hypothesis and analysis. Details of the survey are described in the previous report [11]. In brief, a preliminary internet survey of 30,000 adults drawn from the general public throughout Japan was conducted to identify subtypes of IBS with the Macromill monitor panel (Macromill, Inc., Japan). Identical numbers of males and females from five different age groups (20s, 30s, 40s, 50s and 60–79 years; 3000 subjects each) were screened between October 28 and October 31, 2013. Consequently, of the 30,000 participants, the screened subjects classified as Rome III IBS-C and an equal number of age- and sex-matched non-IBS subjects who were randomly selected as controls were invited to complete a main survey between November 1 and November 4, 2013. In the main survey, IBS-C subjects were asked to answer questions on the degree of anxiety they experienced in their daily lives, the number of bowel movements they had in a week and thoughts about their bowel habits, and their dominant gastrointestinal symptoms and exacerbation factors in their daily lives, such as the circumstances and timing of symptoms and exacerbation. The degree of anxiety was assessed on a 4-point ordinate scale (0, Almost; 1, Often; 2, Sometimes; 3, None). The severity of IBS symptoms such as abdominal pain, abdominal discomfort, and abdominal bloating was assessed on a 5-point ordinal scale (0, Very mild; 1, Mild; 2, Moderate; 3, Severe; 4, Very Severe). The detailed questionnaires were attached in the previous report as additional files [11]. From the view point of feasibility, the sample size was set as for our previous report [11] and no other IBS subtypes except for IBS-C were investigated.

For this study, we further examined the differences between the surrogate Rome IV IBS-C subjects and surrogate Rome IV FBD subjects after publication of our previous report [11]. As detailed in the previous report [11], the Rome III IBS-C subjects were defined as experiencing abdominal pain or discomfort at least two to three days per month in the preliminary survey. Among the Rome III IBS-C subjects, we reclassified those experiencing abdominal pain or discomfort at least one day each week as surrogate Rome III IBS-C subjects. In other words, to be classified as a surrogate Rome III IBS-C subject in this report, subjects defined as having recurrent abdominal pain or discomfort (at least one day per week in the last three months) also needed to be associated with at least two of the following: improvement with defecation, onset associated with a change in stool frequency, and/or onset associated with change in stool form. The diagnosis of IBS was subtyped by the predominant stool pattern: constipation (IBS-C), diarrhea (IBS-D), mixed (IBS-M), or unspecified (IBS-U) [2]. Among the surrogate Rome III IBS-C subjects, we reclassified subjects experiencing abdominal pain in the main survey as surrogate Rome IV IBS-C subjects and subjects not experiencing abdominal pain in the main survey as surrogate Rome IV FBD subjects. Symptoms were quantified and compared between these Rome IV IBS-C subjects and FBD subjects. The two groups were compared using the Mann-Whitney U-test or χ2 test, and associations between the two groups in symptoms and exacerbating factors were evaluated with Kendall’s τ-b. Because the surrogate Rome IV FBD subjects did not have abdominal pain, some statistics regarding abdominal pain were not carried out. We analyzed the association between gender and age and degree of anxiety in these Rome IV IBS-C and FBD subjects using the χ2 test. Age was stratified into two groups, < 50 years and ≥ 50 years. Age ≥ 50 years is considered a risk factor for diagnosing IBS in the Japanese guidelines [8]. Analysis of multiplicity was not carried out in the χ2 test of each symptom. The level of statistical significance was set at a *P*-value of < 0.05. Statistical analysis was assessed by IBM SPSS Statistics.

## Results

**Fig. 1:**
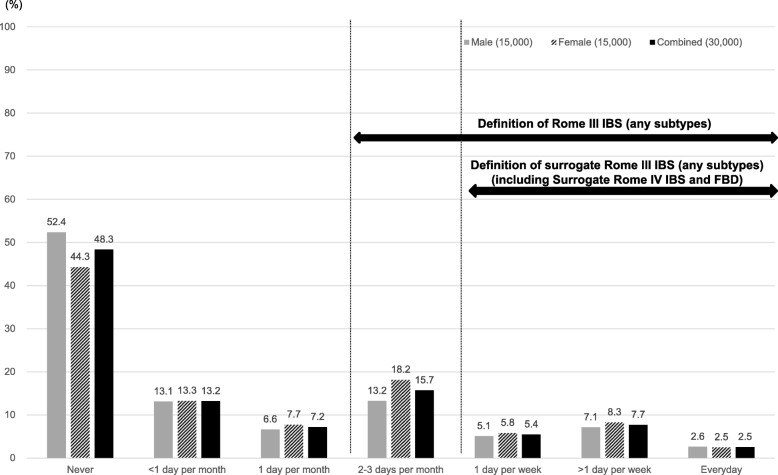
Response among subjects to the question “In the last three months, how often did you have discomfort or pain anywhere in your abdomen?”

A histogram depicting the occurrence of abdominal discomfort or abdominal pain in the general population is shown in Fig. 1.

**Fig. 2:**
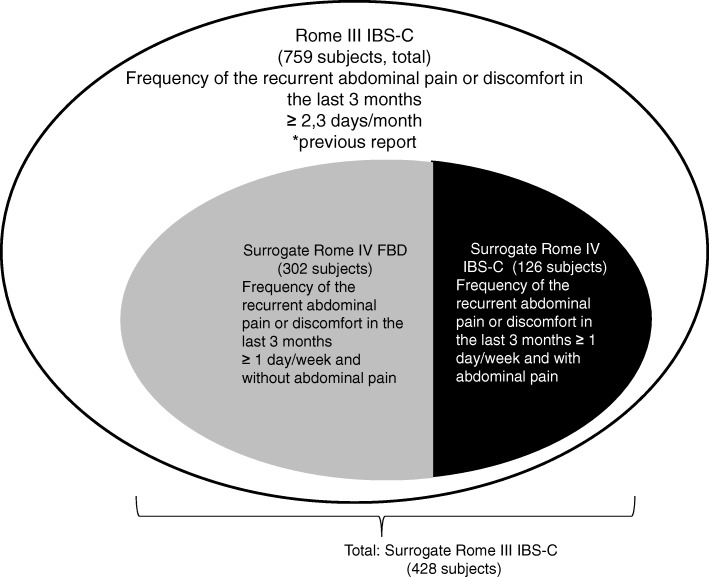
Distribution of Rome III IBS-C and surrogate Rome IV IBS-C/FBD subjects

The demographics of participants in each Rome criteria group are shown in Table 1. Four hundred and twenty-eight (305 female; 71.3%,) surrogate Rome III IBS-C subjects, consisting of 302 (212 female; 70.2%,) surrogate Rome IV FBD subjects and 126 (93 female; 73.8%,) surrogate Rome IV IBS-C subjects, completed the consecutive questionnaires in the main survey. Distribution of Rome III IBS-C and surrogate Rome IV IBS-C/FBD subjects is shown in Fig. 2. The mean age ± standard deviation (SD) of the surrogate Rome IV IBS-C subjects was younger than that of the Rome IV FBD subjects (43 ± 14 vs. 49 ± 14, *p* < 0.001). The surrogate Rome IV IBS-C subjects felt a higher degree of anxiety in their daily lives (p < 0.001) than the Rome IV FBD subjects. There were no significant differences between the surrogate Rome IV IBS-C subjects and Rome IV FBD subjects in the frequency of bowel movements, ideal frequency of bowel movements, and bowel habit, which is considered to be an indicator of health (Table 1).

**Table 1:** Comparison of assumptions about bowel habits between the surrogate Rome IV FBD (not experiencing abdominal pain) and IBS-C (experiencing abdominal pain) subjects

	Total (Abdominal pain or discomfort in the last 3 months ≥ 1 day/week)	Not experiencing abdominal pain	Experiencing abdominal pain	
	Surrogate Rome III IBS-C subjects (*n* = 428)	Surrogate Rome IV FBD subjects (*n* = 302)	Surrogate Rome IV IBS-C subjects (*n* = 126)	*p*-value (Surrogate Rome IV FBD vs Surrogate Rome IV IBS-C)
Female/male (n)	305/123 (71.3/28.7)	212/90 (70.2/29.8)	93/33 (73.8/26.2)	n.s.
Age (mean ± SD, years)	47 ± 14	49 ± 14	43 ± 14	< 0.001
Frequency of bowel movements (median, times/week)	3	3	3	n.s.
Ideal frequency of bowel movements				n.s.
6 times/week or less	92 (21.5)	71 (23.5)	21 (16.7)	
7 times/week	319 (74.5)	221 (73.2)	98 (77.8)	
8 times/week or more	17 (4.0)	10 (3.3)	7 (5.6)	
Considered bowel habit to be an indicator of health				n.s.
None	37 (8.6)	27 (8,9)	10 (7.9)	
Sometimes	100 (23.4)	72 (23.8)	28 (22.2)	
Often	110 (25.7)	86 (28.5)	24 (19.0)	
Mostly	94 (22.0)	66 (21.9)	28 (22.2)	
Always	87 (20.3)	51 (16.9)	36 (28.6)	
Degree of anxiety in daily life				*p* < 0.001
None	97 (22.7)	72 (23.8)	25 (19.8)	
Sometimes	238 (55.6)	176 (58.3)	62 (49.2)	
Often	72 (16.8)	47 (15.6)	25 (19.8)	
Almost	21 (4.9)	7 (2.3)	14 (11.1)	

Data are expressed as n (%) unless otherwise indicated

The degree of anxiety was significantly associated with abdominal bloating (Kendall’s τ = 0.21, *p* < 0.05) and abdominal discomfort (τ = 0.17, p < 0.05) in the surrogate Rome IV IBS-C subjects, but not with the frequency of bowel movements (τ = − 0.10, n.s.). In the surrogate Rome IV FBD subjects, in contrast, the degree of anxiety was not significantly associated with abdominal bloating (Kendall’s τ = 0.09, n.s.), abdominal discomfort (τ = 0.03, n.s.) or the frequency of bowel movements (τ = 0.01, n.s.) (Table 2).

**Table 2:** Associations between the degree of anxiety and GI symptoms of the surrogate Rome IV FBD and IBS-C subjects

	Surrogate Rome IV FBD subjects					Surrogate Rome IV IBS-C subjects				
GI symptoms/Degree of anxiety	None	Sometimes	Often	Almost	Kendall’s τ	None	Sometimes	Often	Almost	Kendall’s τ
Abdominal bloating					0.09					0.21*
No	20	28	7	2		6	6	0	1	
Yes	52	148	40	5		19	56	25	13	
Abdominal discomfort					0.03					0.17*
No	27	58	15	3		6	10	1	1	
Yes	45	118	32	4		19	52	24	13	
Abdominal pain					N/A					N/A
No	72	176	47	7		N/A	N/A	N/A	N/A	
Yes	N/A	N/A	N/A	N/A		25	62	25	14	
Frequency of bowel movement in a week					0.01					−0.10
> 1 time	2	4	2	0		1	2	1	1	
1 time	3	22	3	1		3	7	6	1	
2 times	19	42	12	1		4	22	4	4	
3 times	17	29	6	1		6	9	10	3	
4 times	8	19	6	0		2	4	1	2	
5 times	6	20	5	3		1	6	0	0	
6 times	5	11	3	0		2	3	1	1	
7 times	9	19	9	1		2	5	2	2	
8 times or more	3	10	1	0		4	4	0	0	

*p < 0.05, the Kendall’s τ-b

*GI* gastrointestinal

The association between gender and age and degree of anxiety in these Rome IV IBS-C and FBD subjects is shown in Tables 3 and 4. Female surrogate Rome IV IBS-C subjects and those aged 20–49 years felt a higher degree of anxiety in their daily lives (*p* < 0.05) than the FBD subjects, whereas those male and 50–79 years did not. The association of the combination female and 20–49 years among Rome IV IBS-C and FBD subjects is shown in Table 5. Female Rome IV IBS-C patients aged 20–49 years felt a higher degree of anxiety in their daily lives (p < 0.05) than the Rome IV FBD subjects.

**Table 3:** Comparison of degree of anxiety in daily life between female and male subjects

	Female			Male		
	Surrogate Rome IV FBD subjects (*n* = 212)	Surrogate Rome IV IBS-C subjects (*n* = 93)	*p*-value	Surrogate Rome IV FBD subjects (*n* = 90)	Surrogate Rome IV IBS-C subjects (*n* = 33)	*p*-value
Age (mean ± SD, years)	48 ± 13	42 ± 13	< 0.001	51 ± 15	48 ± 15	n.s.
Degree of anxiety in daily life			< 0.01			n.s.
None	50 (23.6)	17 (18.3)		22 (24.4)	8 (24.2)	
Sometimes	124 (58.5)	45 (48.4)		52 (57.8)	17 (51.5)	
Often	32 (15.1)	21 (22.6)		15 (16.7)	4 (12.1)	
Almost	6 (2.8)	10 (10.8)		1 (1.1)	4 (12.1)	

Data are expressed as n (%) unless otherwise indicated

**Table 4:** Comparison of degree of anxiety in daily life between subjects aged 20–49 and 50–79 years

	Age 20–49 years			Age 50–79 years		
	Surrogate Rome IV FBD subjects (*n* = 139)	Surrogate Rome IV IBS-C subjects (*n* = 86)	*p*-value	Surrogate Rome IV FBD subjects (*n* = 163)	Surrogate Rome IV IBS-C subjects (*n* = 40)	*p*-value
Female/male (n)	101/38 (72.7/27.3)	68/18 (79.1/20.9)	n.s.	111/52 (68.1/31.9)	25/15 (62.5/37.5)	n.s.
Age (mean ± SD, years)	37 ± 9	36 ± 8	n.s.	60 ± 8	60 ± 7	n.s.
Degree of anxiety in daily life			< 0.05			n.s.
None	28 (20.1)	11 (12.8)		44 (27.0)	14 (35.0)	
Sometimes	75 (54.0)	41 (47.7)		101 (62.0)	21 (52.5)	
Often	31 (22.3)	22 (25.6)		16 (9.8)	3 (7.5)	
Almost	5 (3.6)	12 (14.0)		2 (1.2)	2 (5.0)	

Data are expressed as n (%) unless otherwise indicated

**Table 5:** Comparison of degree of anxiety in daily life of female subjects aged 20–49 years

	Female age 20–49 years		
	Surrogate Rome IV FBD subjects (*n* = 101)	Surrogate Rome IV IBS-C subjects (*n* = 68)	*p*-value
Age (mean ± SD, years)	37 ± 8	36 ± 9	n.s.
Degree of anxiety in daily life			< 0.05
None	23 (22.8)	9 (13.2)	
Sometimes	52 (51.5)	31 (45.6)	
Often	22 (21.8)	18 (26.5)	
Almost	4 (4.0)	10 (14.7)	

Data are expressed as n (%) unless otherwise indicated

**Fig. 3:**
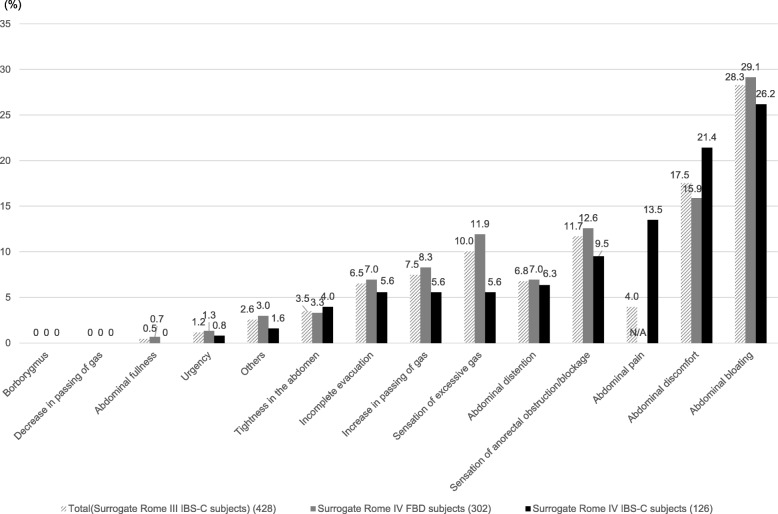
Rate of the most bothersome symptom of the surrogate Rome IV FBD and IBS-C subjects

The most bothersome symptom for the surrogate Rome IV IBS-C subjects was abdominal bloating (26.2%) (Fig. 3), which was the same as that for the corresponding FBD subjects (29.1%). The bloating of the surrogate Rome IV IBS-C subjects was most likely to occur after a meal (60.6%), which was same as that of the FBD subjects (54.5%) (Table 6).

**Table 6:** Occurrence situations of the “most bothersome symptoms” in the surrogate Rome IV FBD and IBS-C subjects

Situations/Symptoms	Total (Abdominal pain or discomfort in the last 3 months ≥ 1 day/week)			Not experiencing abdominal pain			Experiencing abdominal pain		
	Surrogate Rome III IBS-C subjects			Surrogate Rome IV FBD subjects			Surrogate Rome IV IBS-C subjects		
	Abdominal bloating (*n* = 121)	Abdominal discomfort (*n* = 75)	Abdominal pain (n = 17)	Abdominal bloating (*n* = 88)	Abdominal discomfort (*n* = 48)	Abdominal pain (n = N/A)	Abdominal bloating (n = 33)	Abdominal discomfort (*n* = 27)	Abdominal pain (*n* = 17)
On the way to work/school by bus or train	19.8	24.0	0	17.0	18.8	N/A	27.3	33.3	0
At work/school	31.4	36.0	23.5	31.8	27.1	N/A	30.3	51.9	23.5
During a conference presentation/an exam	5.0	12.0	5.9	6.8	4.2	N/A	0	25.9	5.9
After drinking alcohol	0.8	6.7	0	1.1	6.3	N/A	0	7.4	0
After drinking milk	3.3	5.3	11.8	3.4	2.1	N/A	3.0	11.1	11.8
After a meal	56.2	42.7	41.2	54.5	45.8	N/A	60.6	37.0	41.2
On a sightseeing trip	23.1	13.3	0	19.3	14.6	N/A	33.3	11.1	0
On a business trip	3.3	8.0	0	3.4	6.3	N/A	3.0	11.1	0
During time of stress	28.1	28.0	35.3	27.3	22.9	N/A	30.3	37.0	35.3
After taking medication	2.5	1.3	11.8	3.4	0	N/A	0	3.7	11.8
During menstruation (females only)	7.4	10.7	35.3	6.8	4.2	N/A	9.1	22.2	35.3

Data are expressed as frequencies (%)

There was no significant difference in the severity of abdominal bloating and abdominal discomfort between the surrogate Rome IV IBS-C and FBD subjects (Table 7).

**Table 7:** Severity of the “most bothersome symptoms” in the surrogate Rome IV FBD and IBS-C subjects

GI symptoms	0:Very mild	1:Mild	2:Moderate	3:Severe	4:Very Severe	*p*-value (Surrogate Rome IV FBD vs Surrogate Rome IV IBS-C)
Abdominal bloating						
Surrogate Rome IV FBD subjects (n = 88)	0	4.5	28.4	52.3	14.8	n.s.
Surrogate Rome IV IBS-C subjects (n = 33)	6.1	3.0	12.1	54.5	24.2	
Abdominal discomfort						
Surrogate Rome IV FBD subjects (n = 48)	2.1	6.3	41.7	33.3	16.7	n.s.
Surrogate Rome IV IBS-C subjects (n = 27)	0	3.7	33.3	33.3	29.6	
Abdominal pain						
Surrogate Rome IV FBD subjects (n = N/A)	N/A	N/A	N/A	N/A	N/A	N/A
Surrogate Rome IV IBS-C subjects (*n* = 17)	5.9	5.9	23.5	52.9	11.8	

Data are expressed as rate (%)

*GI*, gastrointestinal

**Fig. 4:**
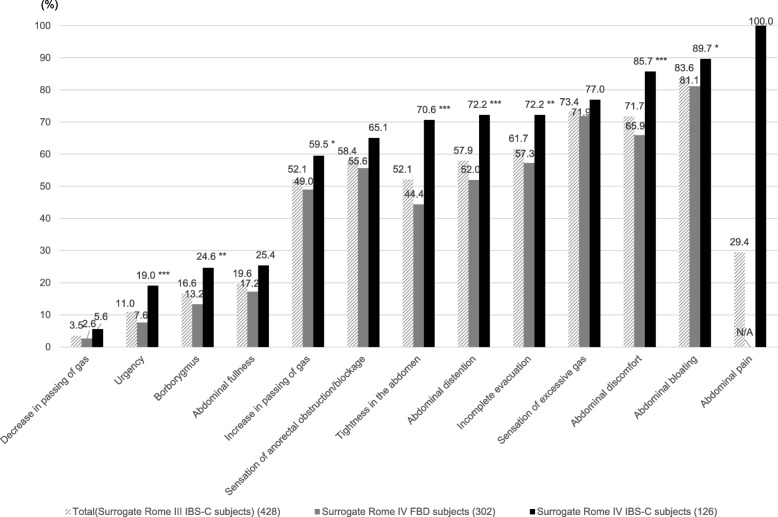
Rate of GI symptoms of the surrogate Rome IV FBD and IBS-C subjects. *P < 0.05, **P < 0.01, ***P < 0.001 vs surrogate Rome IV FBD subjects

The most common symptom other than abdominal pain associated with the constipation of the surrogate Rome IV IBS-C subjects was abdominal bloating (89.7%), which was similar to that of the Rome IV FBD subjects (81.1%). The expression rate of all IBS symptoms reported by the surrogate Rome IV IBS-C subjects was slightly higher than that of the FBD subjects. In particular, the expression rate of all IBS symptoms, except for a sensation of excessive gas, sensation of anorectal obstruction/blockage, abdominal fullness, and decrease in the passing of gas, was significantly higher in the surrogate Rome IV IBS-C subjects than in the Rome IV FBD subjects (*p* < 0.05) (Fig. 4). Analysis of multiplicity was not carried out in the χ2 test of each symptom.

## Discussion

To our knowledge, this is the first report on the influence of the abdominal pain restriction on the diagnosis of IBS-C in the Rome IV criteria. In a simple comparison between Rome III and Rome IV, it is unknown whether this difference reflects the influence of symptomatic frequency or the presence of abdominal pain. However, the advantage of this study is that we were able to extract and analyze the influence of the presence or absence of abdominal pain on the diagnosis of IBS-C.

The surrogate Rome IV IBS-C subjects had greater anxiety than the surrogate Rome IV FBD subjects. The degree of anxiety correlated with GI symptoms in the Rome IV IBS subjects but not in the Rome IV FBD subjects. These results are considered to be in the same direction as other results, including brain imaging findings related to anxiety caused by abdominal pain stimulation [13]; amygdala activation in IBS patients [14]; strong activation of the amygdala when IBS patients are administered the stress-related peptide CRH [15]; remarkable abdominal pain-related activation in IBS patients against anxiogenic stimuli [16]; and the correlation between stressful life events and the exacerbation of GI symptoms in IBS patients but not in FBD patients or healthy controls [17].

We observed that the expression rate of almost all symptoms of the surrogate Rome IV IBS-C subjects was higher than that of the Rome IV FBD subjects, and that the severity of the most bothersome symptoms (abdominal bloating and abdominal discomfort) did not differ between the surrogate Rome IV IBS-C and FBD subjects. Although Rome IV IBS is considered to be mainly a subgroup of Rome III IBS with more serious symptoms [18], these results suggest that Rome IV IBS-C might primarily be a subgroup of Rome III IBS-C with stronger expression of symptoms. Accordingly, the new classification of Rome IV may be more accurate for Japanese IBS-C patients.

Among our findings, we found no substantial difference in the most bothersome symptom (abdominal bloating) between the surrogate Rome IV IBS-C and FBD subjects. Because we assumed that bloating as a Japanese term might encompass “abdominal bloating”, “abdominal distention”, “sensation of excessive gas”, “tightness in the abdomen” and “abdominal fullness”, we surveyed the frequencies of these different terms among IBS-C subjects. Abdominal bloating is associated with decreased QOL and may cause a higher number of physician visits [19]. The IBS symptom severity scale (IBS-SSS) [20], which is widely used to assess the severity of IBS, consists of the items on the severity score for abdominal bloating as well as abdominal pain. However, the presence of abdominal bloating is not necessarily considered a diagnostic criterion for IBS, or for other functional GI disorders except functional abdominal bloating/distension [19]. While there is no consensus on indicators of the efficacy of IBS evaluation among countries, the Food and Drug Administration (FDA) offered guidance for clinical studies of IBS in 2012 [21] and the European Medicines Agency (EMA) announced guidelines for clinical studies of IBS in 2014 [22]. Of these, intensity of abdominal pain and frequency of bowel movements were recommended as the primary endpoints in clinical studies of IBS-C. Although the Pharmaceuticals and Medical Devices Agency (PMDA) in Japan has not announced guidelines for clinical strategies for IBS, abdominal bloating may be one of the most valuable endpoints for Japanese IBS-C patients and should be evaluated in them. In order to address the abdominal bloating of IBS-C patients, therapy that improves not only abdominal bloating but also aids defecation is needed. Because symptoms other than abdominal bloating differ between Rome IV FBD and Rome IV IBS-C patients, there may be differences in therapy for resolving their problems.

Abdominal bloating is considered a key symptom among IBS patients in Asia, and may be an important reason prompting IBS-C patient consultations in Japan [23]. Differences in the interpretation and sensation of abdominal bloating in IBS-C were discussed by the Rome IV experts. In Rome IV, even when speaking the same language, it is not uncommon for patients and doctors to misunderstand each other with regard to symptom reporting. The pictorial version of bloating is considered a good example of how patients can easily respond to visual options that may be difficult to understand or translate from language to language and culture to culture, as with the Bristol stool form scale [24]. In the Asian region, a visual scale of abdominal bloating should be developed for the major features of IBS-C.

IBS-C and functional constipation or chronic constipation are spectrum disorders, which are well conceptualized in the Rome IV criteria. The most bothersome symptom (abdominal bloating) in Japanese IBS-C patients was same as that in Japanese chronic constipation patients [25]. The clinical practice guidelines for chronic constipation in Japan include several opinions that highlight common challenges in the management of chronic constipation and IBS-C [26]. It may be useful in clinical practice to assess abdominal bloating not only for IBS-C but also for chronic constipation.

We previously reported that the most bothersome symptom (abdominal bloating) was most likely to occur after a meal [11]. This result did not change between the surrogate Rome IV IBS-C and FBD subjects. IBS symptoms such as abdominal pain and bloating occur or are exacerbated postprandially in approximately two-thirds of patients [27, 28]. It is possible that the administration of IBS-C treatment before meals will prevent the worsening of abdominal symptoms associated with the anxiety of Rome IV IBS-C patients.

The most bothersome symptom (abdominal pain) of the surrogate Rome IV IBS-C subjects in a previous report was observed mostly after meals, whereas for the Rome III IBS-C subjects it was observed mostly during menstruation [11]. Although it has been reported that the menses of IBS patients is associated with a worsening of abdominal pain [29], this result suggests that this association may be weaker in Rome IV IBS-C than in Rome III IBS-C.

Several limitations of our study warrant mention. First, we used data from an Internet-based survey. Subjects who were interested in their health might have been more likely to participate in this survey. A total of 30,000 samples were collected from a large monitor panel throughout Japan and selected to ensure the same numbers by sex and several age groups. Due to limitations with the Rome III, we could not use the Rome IV diagnostic questionnaire in this survey. Instead, we reclassified the surrogate Rome IV IBS-C and surrogate Rome IV FBD subjects according to the data in Rome III diagnostic questionnaire. Second, although Rome IV IBS-C subjects who were not diagnosed with the Rome III IBS-C criteria might have been present, there were no surrogate Rome IV IBS-C subjects who were not diagnosed with Rome III IBS-C in this report. We do not consider that this result is particularly remarkable because there were few Rome IV IBS subjects who were not also diagnosed as Rome III IBS subjects [30]. Accordingly, the association between age and degree of anxiety in the Rome IV IBS-C subjects and FBD subjects was analyzed because young people have a higher degree of anxiety. However, given the difference in age between the surrogate Rome IV IBS-C and FBD subjects, an effect of age could not completely be ruled out. Moreover, anxiety was not measured using a strictly validated method, albeit that the measurement was considered to be acceptable at the epidemiological level. Furthermore, we did not investigate whether there were subjects with an organic GI disease and/or an other comorbidity, although it has been reported that IBS patients have more somatic/psychiatric comorbidities which may affect their daily lives than non-IBS subjects [31]. Nevertheless, a histogram of the occurrence of abdominal discomfort or abdominal pain in the population in this study is similar to that in the general population after the exclusion of subjects with physician-diagnosed lower gastrointestinal disorders [32]. Finally, no other IBS subtypes except for IBS-C have been investigated. Future research based on the other IBS subtypes is warranted.

## Conclusion

A large population-based Internet survey suggests that female IBS-C patients aged 20–49 years with abdominal pain in Rome IV have more anxiety than those without abdominal pain in Rome III. Changes in the diagnostic criteria from Rome III to Rome IV will better identify candidates for the biopsychosocial approach.

## Abbreviations

**FBD**: unspecified functional bowel disorder
**GI**: gastrointestinal
**IBS**: irritable bowel syndrome
**IBS-C**: IBS with constipation
**IBS-D**: IBS with diarrhea
**IBS-M**: mixed IBS
**IBS-U**: unspecified IBS
**QOL**: quality of life
